# Character strengths, social anxiety, and physiological stress reactivity

**DOI:** 10.7717/peerj.3396

**Published:** 2017-05-30

**Authors:** Tingting Li, Wenjie Duan, Pengfei Guo

**Affiliations:** 1 School of Public Administration and Humanities, Dalian Maritime University, Dalian, Liaoning, P.R. China; 2 Department of Sociology, Wuhan University, Wuhan, Hubei, P.R. China; 3 Hospital (T.C.M.) Affiliated to Southwest Medical University, Luzhou, Sichuan, P.R. China

**Keywords:** Stress, Physiological recovery, Social anxiety, Character strengths, Positive psychology

## Abstract

In this paper, the effects of character strengths on the physiological reactivity to social anxiety induced by the Trier Social Stress Task were reported. On the basis of their scores in the Chinese Virtues Questionnaire, 30 college students were assigned to either high- (*n* = 15) or low-character-strength (*n* = 15) groups. Their psychological stress and physiological data across three laboratory stages (namely, baseline, stress exposure, and post-stress) were collected. Results indicated that individuals with high character strengths exhibited rapid cardiovascular recovery from baseline to post-stress even if high- and low-character-strength groups showed similar patterns of cardiovascular arousal in response to the stress at baseline and stress exposure. These results prove that character strengths are stress-defense factors that allow for psychological and physiological adaptation to stress.

## Introduction

The imbalance between contextual demands and psychological resources leads to psycho-biological stress responses, which affect mental, physical, and social health (Henry & Stephens, 1977). Several empirical studies have been conducted to explore psychological and biological stress-defense mechanisms (e.g., Sladek et al., 2016). Several theories have been proposed to determine the individual characteristics that serve as important psychological resources during stressful situations. The character serves as a schema that explains and processes information toward self, others, and the world. Some researchers have found the similar structure of character. A total of 24 widely valued strengths, organized under six board virtues, were discussed to recognize and cultivate for education and personal development (Park & Peterson, 2009). Three general character strengths have been identified, namely, care, inquisitiveness, and self-control; a total strength can also be computed to reflect the overall level of an individual’s character strengths (Duan et al., 2012; McGrath, 2015). Although these researchers used different types of experiment, methods, and subjects, these factors may overlap; in other words, the VIA and Chinese Virtue Inventory may not capture the full range of character if its factors are limited to caring (cf cooperativeness), self-regulation (cf self-directedness), and inquisitiveness. Another method of measuring character directly is the use of the Temperament and Character Inventory (TCI) to measure the three character traits of Self-directedness, Cooperativeness, and Self-transcendence (Cloninger et al., 1994). In accordance with the Cloninger, Svrakic & Przybeck (1993) model, the content of the character dimensions was constructed to measure individual differences in rational goals and values involving propositional learning. Overcoming some limitations for clinical use, the model maintains strong theoretical and empirical support of previously developed psychobiological models; the model is also widely used to describe individual differences and has received much attention and recognition similar to hierarchical models. Character strengths are protective contributors to a person’s subjective (i.e., satisfaction with life, depression, and quality of life) and physical health (i.e., physical fitness, illness, and symptoms) in various contexts (for a review, see Niemiec, 2013).

The transactional model of stress and coping (Lazarus & Folkman, 1984) states that high character strengths are characterized by an ability to perceive less stress (Duan et al., 2015). A longitudinal study found that the students with high character strengths experience less academic-related stress throughout the semester and flourish after experiencing mild depression symptoms (Duan, 2016). Other retrospective studies revealed that character strengths can promote post-traumatic growth after traumatic events (e.g., earthquake and shooting tragedies) (Duan, Guo & Gan, 2015; Schueller et al., 2015). These studies clarify the psychological stress-defense roles of character strengths. The adaptive properties of character strengths may also be reflected in an individual’s physiological responses to cope with stress.

Allostasis theory (McEwen & Gianaros, 2011) asserts that necessary biological responses immediately appear and subsequently decline during stressful situations. These changes indicate physiological flexibility and adaptiveness during stressful challenges (McEwen, 1998). That is, high character strengths can accelerate psychological recovery after stressful experiences. Systolic blood pressure (SBP), diastolic blood pressure (DBP), and heart rate (HR) are probably the most widely researched cardiovascular indices of threat or challenge (Seery, 2011) as well as critical indices of certain stress-related chronic or cardiovascular diseases (May et al., 2016). Individuals with high levels of openness exhibit better physiological habituation (i.e., low SBP and HR) to recurrent social stress (Lü, Wang & Hughes, 2016). Another study distinguished the physiological reactivity to stress between high- and low-trait-resilient individuals, and the result indicated that the participants with high trait resilience exhibit significant decreases in SBP and DBP (Lü, Wang & You, 2016). Moreover, a study reported the psychobiology of stress in BASE jumpers. In the study, subjects were asked to complete the TCI and salivary cortisol test, and the results showed that a personality profile of psychological resilience called “The Right Stuff” mediated decision making to pursue likely rewards, and was the characteristic of nearly all BASE jumpers; this study has examined the adaptive effect of temperament and character on salivary cortisol reactivity to stress (Monasterio et al., 2016). A commonly used laboratory procedure to induce social stress and anxiety is the Trier Social Stress Task (TSST). This procedure was designed to induce social anxiety through a combination of interview-style presentation followed by a surprise mental arithmetic (Kirschbaum, Pirke & Hellhammer, 1993). Numerous studies have successfully adopted this protocol to cause anxiety-related stress in participants (e.g., Cruess et al., 2015). Allen et al. (2017) conducted a comprehensive review of the TSST principles and practices adopted in studies and concluded the extensive application of TSST across multiple laboratories. Therefore, TSST is a benchmark for the assessment of social stress under laboratory conditions.

In the present study, the standardized procedure of TSST was used to induce anxiety-related stress, and the associations between character strengths and physiological stress reactivity were examined. The participants were divided into high- and low-character-strength groups to explore the differences between the two groups in terms of their physiological recovery from social stress. The hypothesis that high-character-strength individuals will not consider the psychosocial stressors stressful unlike low-character-strength individuals was tested in this study. Kirschbaum, Pirke & Hellhammer (1993) used TSST to describe a protocol for induction of moderate psychological stress in a laboratory setting and evaluated its effects on physiological responses. This study also examined the physiological stress reactivity of individuals with different levels of character strengths in a laboratory setting. The results can enhance the stress-defense role of character strengths from the biological perspective in applied positive psychology. Depersonalization symptoms during a stressful situation have been examined (Hoyer et al., 2013). Therefore, TSST is not only used to activate social anxiety of healthy people to explore their positive personality but also applied to patients to treat their personality disorders in clinical field.

## Methods

### Participants

Participant recruitment was initiated by publishing an advertisement on the university website. Individuals who had a history of serious mental and physical illnesses or were ill at the time of the study were excluded in accordance with the criteria published on the advertisement. Finally, among more than 30 volunteers who signed up, only 30 volunteers conformed to the requirements; they were then asked to participate in the experiment (12 males and 18 females; mean age = 20.10 years old, SD = 1.52). All the participants were required not to consume caffeine and other stimulants 24 h prior to the experiment and not to perform strenuous exercises 2 h before entering the laboratory. The Human Subject Ethics Committees of Southwest University approved this study, and participants signed a written consent. At the end of the experiment, the participants received $8.00 (approximately 50 RMB) as compensation.

### Measures

#### Character strengths

Character strengths were measured using the 96-item self-reported three-dimensional model of character strengths (Duan et al., 2013; Duan et al., 2012) on a 5-point Likert scale (1 = very much unlike me; 5 = very much like me). The Chinese Virtues Questionnaire was used to assess three categories of character strengths, namely, caring, inquisitiveness, and self-regulation, which were identified in previous empirical studies on the basis of VIA classification. The total strength scores were computed to quantify the overall level of individual strength. The coefficients of internal reliability of the total questionnaire (*α* = .96), caring subscale (*α* = .93), inquisitiveness subscale (*α* = .92), and self-regulation subscale (*α* = .90) were good (Duan et al., 2013).

#### State anxiety

State anxiety was measured using the 20-item State Anxiety Inventory (SAI) (Spielberger, Gorsuch & Lushene, 1970). In the study of Shek (1988), the factor structure and reliability of the Chinese version of State Anxiety Inventory was acceptable among 2,150 students. The participants were required to rate the items on a 4-point Likert scale, with 1 for nearly never and 4 for nearly always.

#### Physiological stress responses

SBP, DBP, and HR were measured with an electronic sphygmomanometer (HEM-7051, Omron).

### Procedures

At the participant recruitment stage, the participants were e-mailed the URL of the online character strength measure and were invited to complete it for grouping purposes. The valid data were 30, indicating that 30 participants would take part in the entire experiment. Therefore, the median was used to split them to high-character-strength group (15 individuals; mean = 11.74, SD = 0.79) and low-character-strength group (15 individuals; mean = 10.02, SD = 0.45). The difference in the character strength levels between the two groups was significant (*t* = 7.39, *p* < .001).

All participants were exposed to the study between 3:00 and 4:00 p.m. to eliminate the cyclical changes in physiological reactivity factors (i.e., HR, SBP, and DBP). When the participants arrived at the laboratory, they were given 15 min sitting in a chair to take a rest, and were then administered with a three-stage stress exposure design (i.e., baseline, stress exposure, and recovery). The first, second, and third measurements were used for recording the participants’ reaction in the natural state, under the pressure, and after recovering from stress. First, the participants were requested to complete the SAI for the assessment of the baseline of state anxiety and physiological reactivity (T1). Second, the participants were asked to deliver a public speech and performed serial subtraction in front of a working VCR, and the recorded video would be evaluated by a committee. During the speech, the participants were asked to participate in a mock job interview. During the serial subtraction, they were asked to subtract the number 13 from 45,392 serially as fast and accurately as possible. The SAI and physiological reactivity test was administered the second time after the completion of the public speech and subtraction (T2). Finally, after a 15 min recovery period, the researchers again measured the state of anxiety and physiological reactivity of the participants (T3). Each stage lasted for 10–15 min.

## Results

The descriptive statistics of the psychological and physiological variables at T1, T2, and T3 are shown in Table 1. The differences in these variables between high- and low-character-strength groups were tested by *t*-test. The results are also provided in Table 1. At the baseline, no differences existed in the state anxiety (*t* = 1.76, *p* = .09), HR (*t* = − 0.01, *p* = .99), SBP (*t* = − 1.05, *p* = .30), and DBP (*t* = 1.51, *p* = .14) between the low- and high-character-strength groups. From the baseline (T1) to stress exposure (T2), the paired *t*-test results showed increases in the state anxiety (high-character-strength group: *t* = 6.26, *p* < .001; low-character-strength group: *t* = 8.59, *p* < .001), HR (high-character-strength group: *t* = 6.46, *p* < .001; low-character-strength group: *t* = 14.73, *p* < .001), SBP (high-character-strength group: *t* = 7.92, *p* < .001; low-character-strength group: *t* = 11.90, *p* < .001), and DBP (high-character-strength group: *t* = 7.86, *p* < .001; low-character-strength group: *t* = 4.93, *p* < .001) in both groups. These results implied that the public speech and serial subtraction task effectively increased the participants’ psychological and physiological stress.

**Table 1:** Descriptive statistics of psychological and physiological variables at three time points.

	Total group (*N* = 30)		Low-strength group (*n*_1_ = 15)		High-strength group (*n*_2_ = 15)	
	Mean	SD	Mean	SD	Mean	SD
CS	10.88	1.08	10.02	0.45	11.74	0.79
SA_T1	1.57	0.30	1.66	0.33	1.48	0.26
SA_T2	2.46	0.51	2.73	0.44	2.18	0.41
SA_T3	1.77	0.41	2.00	0.38	1.54	0.29
HR_T1	84.90	12.98	84.87	10.40	84.93	15.51
HR_T2	113.40	15.72	121.00	12.17	105.80	15.51
HR_T3	99.63	18.25	108.53	14.02	90.73	17.99
SBP_T1	115.37	6.47	114.13	6.82	116.60	6.07
SBP_T2	134.93	6.79	138.33	7.03	131.53	4.61
SBP_T3	121.43	8.50	123.53	10.13	119.33	6.14
DBP_T1	74.40	8.92	76.80	10.09	72.00	7.11
DBP_T2	84.30	8.26	88.07	5.98	80.53	8.68
DBP_T3	77.70	10.12	81.87	8.37	73.53	10.24

**Notes.**

CS Character Strengths SA State Anxiety HR Heart Rate SBP Systolic Blood Pressure DBP Diastolic Blood Pressure T1 Time 1 T2 Time 2 T3 Time 3

Mixed-measured 2 (high- and low-character-strength groups) × 3 (T1, T2, and T3) ANOVAs were conducted for state anxiety, HR, SBP, and DBP.

For state anxiety, the main effects of grouping (*F* = 15.83, *p* < .001, ${\eta }_{p}^{2}=0.36$) and time (*F* = 83.82, *p* < .001, ${\eta }_{p}^{2}=0.75$) as well as their interaction effect (*F* = 3.59, *p* = .03, ${\eta }_{p}^{2}=0.11$) were significant. In the low-character-strength group (*F* = 30.19, *p* < .001), the analysis of simple effects indicated that the state anxiety significantly increased from T1 to T2 (1.66 ± 0.33, 2.73 ± 0.44; *p* < .001). Similarly, in the high-character-strength group (*F* = 21.02, *p* < .001), the analysis of simple effects indicated that the state anxiety significantly increased from T1 to T2 (1.48 ± 0.26, 2.18 ± 0.41; *p* < .001). Nevertheless, the state anxiety of the participants in the low-character-strength group significantly increased from T1 to T3 (1.66 ± 0.33, 2.00 ± 0.38; *p* = .02), whereas that of the participants in the high-character-strength group did not (1.48 ± 0.26, 1.54 ± 0.29; *p* = .58).

For HR, the main effects of grouping (*F* = 5.21, *p* = .03, ${\eta }_{p}^{2}=0.16$) and time (*F* = 112.36, *p* < .001, ${\eta }_{p}^{2}=0.80$) as well as their interaction effect (*F* = 12.88, *p* = < .001, ${\eta }_{p}^{2}=0.32$) were significant. In the low-character-strength group (*F* = 33.49, *p* < .001), the analysis of simple effects indicated that the HR significantly increased from T1 to T2 (84.87 ± 10.40, 121.00 ± 12.17; *p* < .001). Similarly, in the high-character-strength group (*F* = 6.49, *p* < .01), the analysis of simple effects indicated that the HR significantly increased from T1 to T2 (84.93 ± 15.51, 105.80 ± 15.51; *p* < .01). Nevertheless, the HR of the participants in the low-character-strength group significantly increased from T1 to T3 (84.87 ± 10.40, 108.53 ± 14.02; *p* < .001), whereas that of the participants in the high-character-strength group did not (84.93 ± 15.51, 90.73 ± 17.99; *p* = .34).

For SBP, the main effects of grouping (*F* = 2.77, *p* = .11, ${\eta }_{p}^{2}=0.09$) were insignificant. By contrast, the main effect of time (*F* = 73.78, *p* < .001, ${\eta }_{p}^{2}=0.73$) and the interaction effect (*F* = 4.20, *p* = .02, ${\eta }_{p}^{2}=0.13$) were significant. In the low-character-strength group (*F* = 33.72, *p* < .001), the analysis of simple effects indicated that the SBP significantly increased from T1 to T2 (114.13 ± 6.82, 138.33 ± 7.03; *p* < .001). In the high-character-strength group (*F* = 29.71, *p* < .001), the analysis of simple effects similarly indicated that the SBP significantly increased from T1 to T2 (116.60 ± 6.07, 131.53 ± 4.61; *p* < .001). However, the SBP of the participants in the low-character-strength group significantly increased from T1 to T3 (114.13 ± 6.82, 123.53 ± 10.13; *p* < .01), whereas that of the participants in the high-character-strength group did not (116.60 ± 6.07, 119.33 ± 6.14; *p* = .19).

For DBP, the main effects of grouping (*F* = 5.96, *p* = .02, ${\eta }_{p}^{2}=0.18$) and time (*F* = 38.03, *p* < .001, ${\eta }_{p}^{2}=0.58$) were significant. By contrast, their interaction effect (*F* = 1.28, *p* = .29, ${\eta }_{p}^{2}=0.04$) was insignificant. In the low-character-strength group (*F* = 6.90, *p* < .01), the analysis of simple effects indicated that the DBP significantly increased from T1 to T2 (76.80 ± 10.09, 88.07 ± 5.98; *p* < .01). Similarly, in the high-character-strength group (*F* = 4.04, *p* = .03), the analysis of simple effects indicated that the DBP significantly increased from T1 to T2 (72.00 ± 7.11, 80.53 ± 8.68; *p* = .01). However, the increases in the DBP levels of the participants in the low-character-strength group (76.80 ± 10.09, 81.87 ± 8.37; *p* = .10) and high-character-strength group (72.00 ± 7.11, 73.53 ± 10.24; *p* = .64) from T1 to T3 were insignificant.

**Figure 1:**
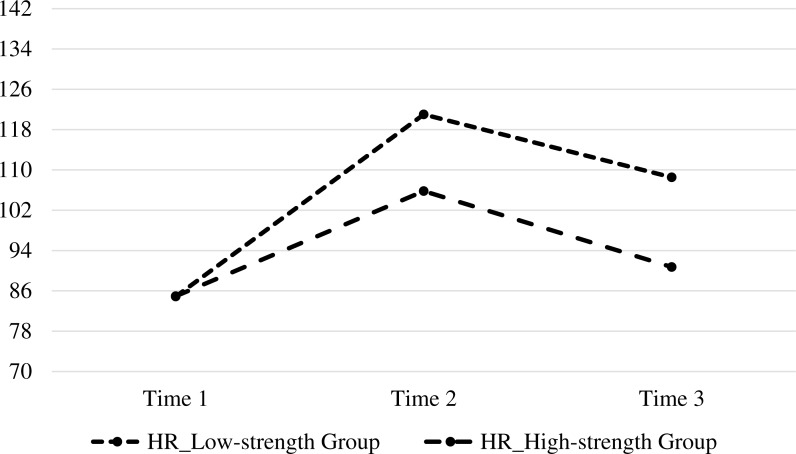
Mean HR reactivity to stress in low-strength and high-strength groups at Time 1, Time 2, and Time 3.

**Figure 2:**
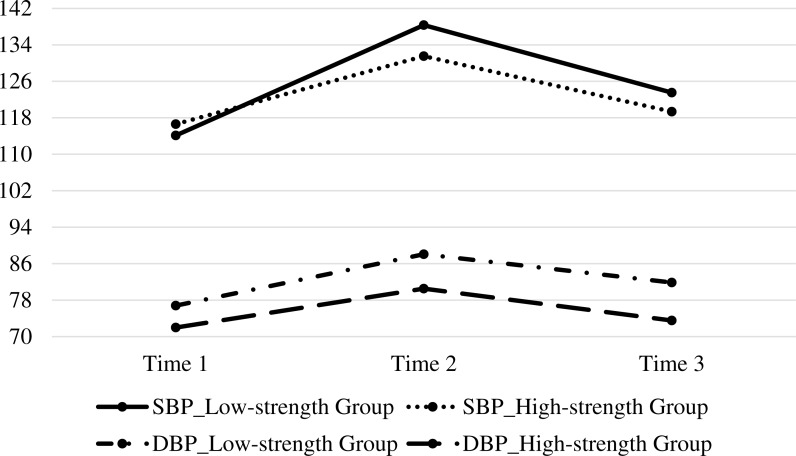
Mean SBP, and DBP reactivity to stress in low-strength and high-strength groups at Time 1, Time 2, and Time 3.

These results are visualized in Figs. 1 and 2.

## Discussion

Individuals with high character strengths are likely to perceive less stress and demonstrate healthy psychophysiological responses (Duan et al., 2015; Harzer & Ruch, 2015; Li & Liu, 2016). HR, SBP, and DBP are believed to be highly physical stress tests in comparison with state anxiety, which is a mental stress test. The former tests are objective indicators, whereas the latter is a self-appraisal on the severity of stress. The relationship between psychological phenomenon and stress-related physiological indexes has been extensively explored, and previous results showed that the change in emotion and stress can lead to different changes in the physiological indexes. However, given the instability of the body state, psychological indexes are also used as an effective complement for objective indicators to observe the subjective psychological activity (Quan, Zhang & Hu, 2011). The present study aimed to examine whether responses to physical and mental stress tests are influenced by personality, and identify possible personality traits related to increased reactivity. The present study suggested that, compared with participants with low character strengths, participants with high character strengths exhibited psychological and physiological adaptation to stress; as a result, the high-character-strength participants had low state anxiety, HR, SBP, and DBP. This study extends previous studies on character strengths and stress. Individuals with high character strengths were found to exhibit faster cardiovascular recovery from stress from T1 to T3 compared with those with low character strengths, even if both groups showed similar patterns of cardiovascular arousal in response to the stress at T1 and T2 (Fig. 1). In general, the high-character-strength group manifested better physiological recovery from stress than the low-character-strength group did. The four measures (i.e., state anxiety, HR, SBP, and DBP) were rapidly recovered with high character strength. Differences were observed among these stress indicators. Accordingly, which indicators were highly sensitive to stress reaction were determined. The results showed that people in high-strength group presented better recovery for SBP than people in low-strength group, whereas both groups exhibited good recovery for DBP. In other words, the recovery for SBP of people in low-strength group was slow. Therefore, their SBP was highly sensitive to stress. When a similar reaction called White-Coat Hypertension (i.e., patients’ nervousness due to clinic environment) is serious and depressive for patients, much catecholamine is generated in the blood, peripheral vascular contraction occurs, and resistance increases, thereby increasing SBP (Franklin et al., 2013). In the present study, the SBP of people with low strength was sensitive to stress and recovered gradually, whereas their DBP quickly returned to normal.

Character strengths are stress-defense factors. Avey (2012) found that wisdom strength is negatively related to the reported stress among employees. Furthermore, wisdom strength provides participants with coping strategies to present adaptive responses to stress. Another study identified intellectual, emotional, and interpersonal strengths as coping-related components in a work-stress area, and these strengths significantly mediate the negative effect of stress on job satisfaction (Harzer & Ruch, 2015). Papousek et al. (2010) examined the influence of trait positive affect on cardiovascular stress responses and found that a high trait positive affect is associated with complete cardiovascular and subjective post-stress recovery. These studies pave the way for a new research area on the role of trainable character strengths in coping with stress.

Character strengths reflect individuals’ positive pattern of emotions, thoughts, and behaviors (Park & Peterson, 2009). They highlight positive cognitions to explain internal and external information. Cognitive theories imply that cognition bias plays an important role in anxiety (Bar-Haim et al., 2007). Fredrickson’s (2001) broaden-and-build theory suggests that broadening at the cognitive level mediates undoing at the cardiovascular level. Resilient individuals, who are characterized by positive cognition, positive emotion, or positive qualities, exhibit rapid cardiovascular recovery following a negative emotion. Therefore, these positive patterns can broaden people’s momentary thought–action repertoires and can build enduring personal resources, including physical, social, and psychological resources, to combat stress or other negative influences. In the present study, high-character-strength participants exhibited stress along with increased HR, SBP, and DBP; however, they had the ability to undo the lingering cardiovascular reactivity. In practice, a character strength enhancement program may be developed and integrated with biofeedback to help participants train their character strengths and cope with various types of stresses. Participants may be asked to observe their HR and blood pressure in stressful situations and then asked to recall how their personal character strengths can be used in difficult times and observe the changes in their physiological indices. A visual biofeedback may be developed to enable participants to use their strengths intentionally by means of visual stimulation; consequently, this mechanism can change the autonomic nerve impulses, HR, and blood pressure and ultimately train or reinforce character strengths to overcome stress.

Consistent with previous studies (e.g., Lü, Wang & You, 2016), the present study showed that HR and BP are reliable and effective indicators in explaining differences in responses to stress. In the clinical field, a stressful environment induces sympathetic nerve excitability, rapid heartbeat, accelerated endovascular blood flow speed, increased blood pressure, and increased heart contraction force (Chalmers et al., 2014). These typical manifestations are recognized as risk factors of left ventricular hypertrophy (Burns et al., 2016). Accordingly, the decreases in the HR and BP of high-character-strength individuals despite social stress and anxiety can reflect differences in cardiovascular disease risks. Therefore, participants with high character strengths may have good physical health. However, further study should be undertaken to explore the assumption.

The study has significance for patients’ rehabilitation, especially for cardiovascular patients who often produce negative cognition and emotion that are harmful to heart and blood vessel; this condition not only affects the clinical treatment effect but also decreases quality of life in these patients. Patients can use their strengths to regulate the HR and blood pressure with the aid of visual biofeedback. Humor, hope, and gratitude strengths can help reduce the tension, anxiety, and chronic pain of cardiovascular patients (Forster & Owen, 2012; Ghandeharioun et al., 2016; Lackner et al., 2014). Therefore, the visual biofeedback may serve as an effective complementary method to promote patients’ recovery. Moreover, it provides understanding on the effects of positive personality on physiological reactivity to stress.

## Limitations and Conclusions

Several limitations of this study should be considered when interpreting the current findings. First, the sample size and type were limited. The entire sample was divided into high- and low-character-strength groups; as a result, the effects of the specific character strengths (i.e., caring, inquisitiveness, and self-control) were not emphasized. Future studies can recruit a large number of participants and examine the specific stress-defense role of the primary character strengths in stressful situations. Second, a single-stress task of TSST was adopted in this study. As a result, the habituation trend of character strengths was unexplored. Subsequent studies may employ a recurrent-stress task to expand the findings. Third, other factors, such as gender and age, need to be considered in future studies, given that gender and age were not controlled in the present study because of the small sample size. Allen et al. (2017) reported that TSST reveals gender and age differences in neurobiological responses to stress. Therefore, more studies should be conducted to examine the mediators and moderators in the relationship between character strengths and stress reactivity.

The present study results suggest the potential value of individuals’ character strengths to explore positive personality resources for stress situation. Specifically, the study results demonstrate individuals with high character strengths exhibited rapid cardiovascular recovery from baseline to post-stress. Such results may lead to a new insight or complementary method to promote cardiovascular patients’ recovery. This study provides initial empirical evidence that individuals’ character strengths are stress-defense factors that allow for psychological and physiological adaptation to stress.

##  Supplemental Information

10.7717/peerj.3396/supp-1 Data S1 Dataset Raw data

## Additional Information and Declarations

### Data Availability

The following information was supplied regarding data availability: The raw data has been supplied as Data S1.
